# Intraocular lens power estimation for future emmetropia in pediatric cataract surgery

**DOI:** 10.5935/0004-2749.20220036

**Published:** 2022

**Authors:** Antonio Carlos Lottelli

**Affiliations:** 1 Division of Ophthalmology, Surgical Specialties and Anesthesiology Department, Faculdade de Medicina de Botucatu, Universidade Estadual Paulista “Júlio de Mesquita Filho”, Botucatu, SP, Brazil

**Keywords:** Cataract, Biometry/methods, Emmetropia, Axial length, eye, Lenses, intraocular, Child, Catarata, Biometria/métodos, Emetropia, Comprimento axial do olho, Lentes intraoculares, Criança

## Abstract

**Purpose:**

Creating models, in pediatric cataracts, to estimate kerotometry and axial length values at future ages, based on kerotometry and axial length measured at surgery, to estimate the intraocular lens power for emmetropia in future ages.

**Methods:**

Eyes with bilateral cataract and kerotometry and axial length measured at surgery and at least one postoperative examination with kerotometry and axial length measurements, were considered for this study. The models to estimate future kerotometry and axial length values were created considering (1) kerotometry and axial length measured at surgery, (2) the average slope of kerotometry and axial length logarithmic regression created for every single eye and (3) age at surgery. The intraocular lens for future ages can be estimated using these values in third generation formulas. The estimation errors for kerotometry, axial length and intraocular lens were also calculated.

**Results:**

A total of 57 eyes from 29 patients met the inclusion criteria. The average age at the surgery and follow-up was 36.96 ± 32.04 months and 2.39 ± 1.46 years, respectively. The average slope of logarithmic regression created for every single eye were -3.286 for kerotometry and +3.189 for axial length. The average absolute estimation errors for kerotometry and axial length were respectively: 0.61 ± 0.54 D and 0.49 ± 0.55 mm, and for intraocular lens using SRK-T, Hoffer-Q and Holladay I formulas were: 2.04 ± 1.73 D, 2.49 ± 2.10 D and 2.26 ± 1.87 D, respectively.

**Conclusions:**

The presented models could be used to estimate the intraocular lens power for emmetropia at future ages to guide the choice of the intraocular lens power to be implanted in pediatric cataract.

## Abstract (pt)

**Objetivo:**

Criar modelos, em catarata pediátrica, para estimar valores futuros de ceratometria e comprimento axial, com base na ceratometria e no comprimento axial medidos na cirurgia, para previsão do poder da lente intraocular para emetropia em idades futuras.

**Métodos:**

Olhos com catarata bilateral, ceratometria e comprimento axial medidos na cirurgia e pelo menos um exame pós-operatório com medidas de ceratometria e comprimento axial foram considerados para este estudo. Os modelos para estimar futuras ceratometrias e comprimentos axiais foram criados considerando (1) ceratometria e comprimento axial medidos na cirurgia, (2) a inclinação média da regressão logarítmica da ceratometria e comprimento axial criada para cada olho e (3) a idade na cirurgia. A lente intraocular para emetropia em idades futuras pode ser estimada usando esses valores em fórmulas de terceira geração. Os erros de estimativa da ceratometria, comprimento axial e poder da lente intraocular, usando os modelos, também foram calculados.

**Resultados:**

57 olhos de 29 pacientes preencheram os critérios de inclusão. A idade média na cirurgia e acompanhamento foram de 36,96 ± 32,04 meses e 2,39 ± 1,46 anos, respectivamente. A inclinação média da regressão logarítmica criada para cada olho foi de -3.286 para ceratometria e + 3.189 para o comprimento axial. Os erros médios de estimativa absoluta para ceratometria e comprimento axial foram respectivamente: 0,61 ± 0,54 D e 0,49 ± 0,55 mm, e para o poder da lente intraocular usando as fórmulas SRK-T, Hoffer-Q e Holladay I foram: 2,04 ± 1,73 D, 2,49 ± 2,10 D e 2,26 ± 1,87 D, respectivamente.

**Conclusões:**

Os modelos apresentados podem ser utilizados para estimar o poder da lente intraocular que levaria a emetropia em idades futuras e orientar a escolha do poder da lente intraocular a ser implantada na catarata pediátrica.

## INTRODUCTION

The primary intraocular lens (IOL) implant has been more used with the surgical technological improvement^(1)^. However, besides the technical difficulties, performing the IOL implantation in small eyes is still a challenge due the inflammatory activity and the choice of IOL power.

Corneal flattening^(2)^ and the decrease in crystalline refractive power from approximately +34.4 diopters (D) at birth to approximately +18.8 D in adulthood^(3)^ compensate the axial length (AL) increase in order to maintain emmetropia. Thus, normal eyes may develop low myopia with growth, but pseudophakic eyes will present higher myopia because the dioptric power of implanted IOL remains unchanged^(3)^, if the IOL generates emmetropia at the time of surgery^(4)^.

Therefore, some authors recommend subtracting 20% power in infants (<8 months of age) and 10% power in children between two and three years of age to minimize future myopia^(4)^. Others estimate the AL growth, applying the logarithmic models in the postoperative aphakic refraction *vs.* age, to verify the myopization rate^(5)^. Estimations based on refraction have been used for the past 20 years, because the postoperative refraction is easily obtained, but it is an indirect way to estimate the eye grow. Recent publications shows the advantage of using serial measurement of AL to estimate the evolution of AL with the age to better verify the future myopization^(6,7)^.

This study aims to create models in pediatric cataract to estimate not only AL, but also keratometry (K) values at future ages, based on K and AL measured at surgery, to estimate the IOL power for emmetropia in future ages.

## METHODS

The local Ethics Committee approved this retrospective study from the Protocol for Primary Intraocular Lens Implant for Treatment of Congenital and Developmental Cataract that sequentially included children (age from 0 to 12 years old) with bilateral and unilateral congenital or developmental cataract. The research followed the tenets of the Declaration of Helsinki and that informed consent was obtained from the parent(s) or legal guardian(s), after explanation of the nature and possible consequences of the study.

Only eyes with bilateral cataract and K and AL measured at surgery and one or more postoperative examination with K and AL measurements, at least three months after surgery in patients operated younger than six months old, and at least six months after surgery in patients operated older, were considered for this study. Eyes with glaucoma or glaucoma suspicion were excluded, according to previously published parameters by the Infant Aphakic Treatment Study^(8)^.

At the Protocol, eyes with horizontal corneal diameters smaller than 10 mm or other ocular abnormalities were excluded. Children with congenital cataract, diagnosed during the first weeks of life, were operated on between the 5th and 12th weeks of life, and the second eye was operated on one to two weeks after the first eye or in the same day surgery.

After general anesthesia and before the surgery, automated refraction and K measurements (Retinomax K-plus 2^®^, Righton, Tokyo, Japan), tonometry (Tono-Pen XL^®^, Reichert^®^ Technologies, Buffalo, USA), and immersion ultrasound biometry and pachymetry (OcuScan RxP^®^, Alcon, Fortworth, USA) with AL and anterior chamber depth (ACD) measurements were performed.

At the surgery, a single-piece hydrophobic acrylic intraocular lens was implanted (Alcon *AcrySof*^®^ *IQ, Alcon, Fortworth*) in the bag. The IOL power was calculated using Hoffer-Q formula (ACD constant= 5.37) for an immediate hyperopia to minimize myopia in adulthood; the amount of hyperopia was guided by a table according to age^(9)^. In all patients, the posterior capsule was opened, and an anterior vitrectomy was performed via *pars plana/plicata*.

In children younger than one year, an examination under narcosis at the operating room was scheduled every three months during the first year of life. In children older than one year, the examination was scheduled every six months. In the examination under narcosis, automated refraction and K measurements, tonometry, and immersion ultrasound biometry and pachymetry with AL and pseudophakic ACD measurements were performed using the same devices used at surgery.

Collaborative children older than four years of age were examined in the office without narcosis. Among these children, K and automated refraction measurements were taken using an automated tabletop refractometer and keratometer (Potec PRK-6000^®^, Potec, Daejon, Korea), and AL and ACD measurements were taken using an optical biometer (IOL Master 500^®^, Zeiss, Jena, Germany). IOP was measured using a Goldman tonometer coupled with a slit-lamp.

### Step 1: Creating an estimation model for K and AL

Assuming that K flattening and AL growth follow a logarithmic (log) model according to the increase in age^(10-13)^, a logarithmic regression (*y* = *a* + *b* x *log* age) was calculated for every single eye using two or more measurement in different ages, where *“y”* is the variable (K and AL), *“a”* is the intercept or the point where the graph crosses the *“y”* axis and corresponds to age “zero”, and *“b”* is the slope and denotes the velocity that the variable changes according to the *log*_10_ age. This could be positive, if the variable increases with age (AL), or negative if it decreases (K). Through the linearization process, the logarithmic regression can be transformed into a straight line, changing the scale of the axis-x from linear to logarithmic, making possible to obtain the regression using two (or more) points (i.e., two or more values of K and AL observed at different ages)^(7)^.

The average logarithmic regression for K and AL was considered the logarithmic regression formed by the average intercept (the average of the *“a”* values) and the average slope (the average of the *“b”* values) from the logarithmic regression of every single eye for AL and K.

In order to consider the initial values of K (K_In_) and AL (AL_In_) at the surgery age (A_In_) in the estimation model, the formula *y final* = *y initial* + *b* x *log*_10_ (age final/age initial) was used^(13)^. This formula basically applies the slope “*b*” to the initial values of K and AL to estimate their future values (K_F_ and AL_F_). The slope “*b*” is actually the rate of “K flattening” or “AL growth,”.

### Step 2: Calculating the absolute estimation error of K and AL

The difference between the last K and AL measurements (i.e., the last measurement taken) and estimated K_F_ and AL_F_ values for the age of the last measurements (A_F_), using the estimation model, was calculated for every single eye to determine the average absolute estimation error of the models.

### Step 3: Estimating the IOL and its absolute estimation error for the age of the last measurement

The IOL for emmetropia for the age of the last measurements (A_F_) was estimated for every single eye using K_F_ and AL_F_ estimated in the step 2 and the SRK/T, Hoffer Q and Holladay I formulas. An IOL was also calculated using SRK/T, Hoffer Q and Holladay I formulas, and K and AL values of the last measurements. The difference between the calculated and estimated IOL was verified to determine the average absolute estimation error for the IOL.

## RESULTS

Ninety-four eyes of children with bilateral cataract were operated on a period of 5.5 years, by the same surgeon (ACL). A total of 57 eyes (29 patients) met the inclusion criteria. In one patient only one eye met these criteria. Not all exams were performed on the scheduled dates due to no-show, impossibility to perform the narcosis or others, in these cases a new exam was scheduled for a new date as soon as possible. A total of 238 K and AL measurements were obtained, 57 at surgery, 145 under narcosis, and 36 at the office. The sample characteristics according to the age at the surgery, number of observations performed per eye, age at the last observation and follow-up time are shown in table 1.

**Table 1:** Sample characteristics according to the age at the surgery, number of observations performed per eye, age at the last observation and follow-up

Age at surgery (months)	Number of eyes	Average age (months)	Number of observations	Last visit age (months)	Follow-up (years)
0-6	16	3.29 ± 1.43 (1.44-5.32)	5.25 ± 2.24 (2-8)	31.56 ± 19.20 (7.92-54.24)	2.36 ± 1.60 (0.40-4.35)
6-24	8	10.56 ± 2.88 (6.44-14.50)	3.75 ± 1.39 (2-5)	42.60 ± 24.00 (17.52-66.96)	2.66 ± 1.83 (0.63-4.68)
24-48	13	36.72 ± 6.00 (28.8-44.76)	4.65 ± 2.47 (2-8)	63.84 ± 15.60 (42.96-85.32)	2.26 ± 1.52 (0.98-4.45)
48-72	11	63.96 ± 3.48 (60.12-71.04)	3.27 ± 1.25 (2-5)	87.72 ± 15.72 (71.40-116.88)	1.98 ± 1.34 (0.54-4.25)
> 72	9	88.20 ± 13.92 (71.12-111.00)	3.78 ± 1.39 (2-5)	122.76 ± 21.60 (79.20-149.52)	2.88 ± 0.99 (0.59-3.70)
**Total**	**57**	**36.96 ± 32.04 (1.44-111.00)**	**4.28 ± 2.00 (2-8)**	**65.64 ± 36.84 (7.92-149.52)**	**2.39 ± 1.46 (0.40-4.68)**

The average linear regression for K and AL, and the estimation models of K and AL (Step 1) are found in table 2.

**Table 2:** Average logarithmic regression and estimation model for keratometry (K) and axial length (AL) as a function of age

Variables	Average logarithmic regression	Estimation model
K (D)	*K= 53.966 - 3.286 x log_10_ age*	K_F_= K_In_ -3.286 *x log*_10_ *age* (A_F_/A_In_)
AL (mm)	AL= 10.862 + 3.189 x *log_10_ age*	*AL*_F_= *AL*_In_ +3.189 *x log*_10_ *age (A*_F_*/A*_In_)

*K*_F_= final K; *K*_In_= initial K; *log*_10_= logarithm in base 10; *A*_F_= final age; *A*_In_= initial age; *AL*_F_= final AL; *AL*_In_= initial AL.

The average absolute estimation errors, standard deviation (SD), and 95% confidence intervals (CI) calculated for K and AL were 0.61 ± 0.54 (0.46, 0.76) D and 0.49 ± 0.55 (0.34, 0.61) mm, respectively (Step 2).

The average absolute estimation error between the calculated and estimated IOL, SD, 95% CI, using SRK-T, Hoffer-Q and Holladay I formulas, are shown in table 3 (Step 3).

**Table 3:** Average absolute estimation errors with standard deviations (SD) and 95% confidence interval (CI) of the IOL power (D) in the age of the last measurement, using SRK/T, Hoffer Q and Holladay I formulas

Formula	Average error (D), SD and 95% CI
SRK/T	2.04 ± 1.73 (1.59, 2.49)
Hoffer Q	2.49 ± 2.10 (1.95, 3.04)
Holladay I	2.26 ± 1.87 (1.77, 2.74)

## DISCUSSION

The models described estimates K and AL with average absolute error of 0.61 D and 0.49 mm, and IOL with average absolute error of 2.04 D, 2.49 D and 2.26 D using SRK/T, Hoffer Q and Holladay I, respectively. These results were obtained applying the models to the same sample that generated them.

Only bilateral congenital/ developmental cataract eyes were selected in the present study, although most previous studies have included eyes with unilateral cataracts^(5,7,10-17)^. Children’s eyes with unilateral cataracts are subjected to other abnormalities that might affect growth; they are frequently smaller than fellow normal eyes^(10)^ and often associated with alterations including persistent fetal vasculature^(18)^. Another important fact is that these eyes often develop amblyopia, which may influence the posterior segment growth^(18,19)^. Eyes with glaucoma, which can also affect eye growth^(14-17)^, were excluded. As the analyses involve logarithmic regression in a longitudinal observation, the use of both eyes do not affected them^(6,14)^.

In the literature, two approaches may be considered to evaluate eye growth: each eye being denoted by a point on the K and AL scatterplots as a function of the age, disregarding the particular dynamic growth of each eye^(10-13)^, or considering the dynamic growth of each eye separately, using serial measurements taken during the patients’ growth to create linear^(6)^ or logarithmic regression^(7,14-17)^. The regressions allows to use eyes operated on at different ages, with different follow up, and different number of measurements within the same sample^(5)^. In the sample of the present study there are eyes operated on from 44 days old to nine years old, with follow up from 4.8 months (0.4 years) to 4.68 years, and eyes measured from two to eight times each (Table 1).

A new exam with at least three months after surgery was accepted as an inclusion criterion for patients operated on younger than six months old, whereas for patients operated on older than six months old, the required time interval for a new exam was at least six months. This is due to the fact that K flattens and Al grows quickly in the early ages, and using the log model a few months in the first year could represent a larger segment in the x-axis, which corresponds to the age, than the years in older ages^(7)^. Despite that, the shortest follow-up in the study was 4.8 months.

The logarithmic regression has been used for a long time but with serial measurements of aphakic or pseudophakic refraction. Refraction is an indirect way to access eye growth, but it is an easily available datum in the clinical practice^(5,14-17)^. Only recently, with the advent of autokeratometers and especially optic biometers, serial measurements of K and AL have become available for children. It is possible to use these devices around three or four years old, when the children start to be collaborative. In the sample of the current study, for children under four years old, the K and AL measurements were performed under narcosis, using in the operation room different devices from those utilized in the office. Keratometric measurements taken under narcosis using a keratometer of the handheld automated refractor might be a source of imprecision regarding the lack of gaze fixation^(20)^. Outpatient measurements were taken using the keratometer of a tabletop automated refractor, K values from handheld and tabletop keratometers are comparable^(21)^. Regarding immersion ultrasound and optic biometry, studies on adults show a strong correlation between them^(22)^. although a study on children showed that the AL values measured using an optical biometer are 0.1 mm lower in average than those measured using an immersion biometer^(23)^.

In the first study considering the dynamic growth of the AL (i.e., considering the AL growth of every single eye separately using serial measurements during the children growth) the authors used a multivariable analysis to estimate AL values in the future for patients older than two years old, based on a measured AL at surgery (baseline value), and emphasized the importance to use a baseline value and not only the age in this estimation^(6)^.

The present study uses models that are similar to the recently published one using eyes with bilateral and unilateral pediatric cataract to estimate future values of AL^(7)^, but it also presents, for the first time, as far as we know, an estimation model created for K values. At the published model for AL, the age was corrected adding 0.6 years to correct the asymptotic effect of the log curve around the birth^(7)^; however, this correction was not made in the present study. Therefore, it is necessary to compare them in a future study to evaluate the advantage of this correction.

The regularly used methods to guide the choice of the IOL that will be implanted in a child, estimate an IOL value to leave a residual hyperopia according to age, for example, leaving a child +6.0 D at the age of one year old, expecting that this child will be emmetrope at adulthood^(9)^. However, the IOL formulas were developed for adult eyes and even the most recent formulas are inaccurate for calculation in children^(24-26)^, so the estimation of +6.0 D for a one-year-old child already has an error due to the imprecision of the formulas at this age. Because the method proposed in this study uses future estimation of K and AL values (around or at adulthood) in the formulas, this source of error should be minimized or eliminated.

Application:

To estimate, for example, the IOL to be implanted in a two-month-old (1/6 years) baby with K= 50.0 D and AL= 17.0 mm at surgery, for emmetropia at the age of 18 years old, K and AL are estimated for that age and after used in a third-generation formula as follows:

*K_F_*= *K*_In_ -3.286 *x log*_10_ *age (A*_F_*/A*_In_) K_F_= 50 -3.286 x log_10_ (108)

K_F_= 43.31 D

*AL*_F_= *AL*_In_ +3.189 *x log*_10_ *(A*_F_*/A*_In_) AL_F_= 17 + 3.189 x log_10_ (108)

AL_F_= 23.48 mm

Using K= 43.31 D and AL = 23.48 mm with the SRK-T formula (A constant = 118.7), for example, the IOL power estimation for emmetropia at 18 years old is +21.19 D. (These models and updates can be incorporated into a “Pediatric Calculator” to facilitate estimates).

It does not mean that this study has been advocating an +21.0 D for a two-months-old baby or this IOL power for a baby with same K and AL values at the same age; it is only a guide to help surgeons choose the best IOL power, taking into account other individual factors like unilateral or bilateral cataract, refraction status of the other eye, possibility to use contact lens or not among others, because having a small refractive error in adulthood is good, if possible, but the most important is providing conditions for a good visual development.

It is estimated that several factors like the presence of the IOL, gender, race, age at surgery, and final visual acuity, among others, could influence the eye growth, but the studies are controversial^(7,16,17,27)^. In a multivariable model to predict postoperative AL in children older than 2 years old, only the patient’s baseline age and age at follow-up were considered significant for this prediction^(6)^. This can probably be better elucidated when a bigger number of AL and K measurements are available.

Regarding the absolute estimation errors of the models, they were calculated using the same sample that generated them and, therefore, tending to generate lower errors than those that would be generated with different samples. The initial values of K and AL were also taken at different ages (from 1.44 to 111 months) to estimate future K and AL values at different ages (from 7.92 to 149.52 months) as well (Table 1). The ideal would be to have a different and homogeneous sample to test the models, for example, if all measured values were obtained from eyes at two months old and at 18 years old, but this kind of sample is not available. Taking those into account, the average error was 0.61 for K and 0.49 for AL, which can generate an error around 0.55 D only for K and 1.23 D only for AL in the IOL calculation, if the SRK formula (IOL = A constant - 0.9K - 2.5AL) is considered, and multiplying the K error by 0.9 and AL error by 2.5. Using the estimated values (K_F_ and AL_F_), the IOLs for emmetropia were calculated with third generation formulas to better demonstrate the impact of the errors in the IOL values. It results in errors from 2.04 D to 2.49 D. Only third generation formulas can be used with the available K and AL; in order to use new formulas, other variables are needed. We cannot identify factors that could interfere in the errors like age or K and AL values, It seems to be random, but further studies are necessary to explore it.

Using such varied sample is a limitation of the present study, although the logarithmic regression allows this. An ideal situation would be to have all eyes operated on at the same age, with measurements taken at the same follow-up ages and with a long follow-up, preferably until adulthood; however, this is impossible in real conditions and even samples like in this study, with serial measurements after surgery, are rare. The use of different devices to perform K and AL measurements and the small sample involving patients from a limited region are the other limitations of the study.

Pediatric cataract is relative rare condition, a task force involving surgeons around the world could contribute to broaden the samples and create more accurate models to help the hard decision-making of the IOL power that should be implanted in pediatric cataracts.

In conclusion, this paper presents a model to estimate K and AL values for future ages, based on K and AL measured at surgery. These K and AL values can be used in third-generation formulas to guide the choice of the IOL power to be implanted in pediatric cataract.
